# The Reticular Cell Network: A Robust Backbone for Immune Responses

**DOI:** 10.1371/journal.pbio.2000827

**Published:** 2016-10-11

**Authors:** Johannes Textor, Judith N. Mandl, Rob J. de Boer

**Affiliations:** 1 Department of Tumor Immunology, Radboud Institute for Molecular Life Sciences, Nijmegen, The Netherlands; 2 Department of Physiology, McGill University, Montreal, Québec, Canada; 3 Theoretical Biology, Utrecht University, Utrecht, The Netherlands

## Abstract

Lymph nodes are meeting points for circulating immune cells. A network of reticular cells that ensheathe a mesh of collagen fibers crisscrosses the tissue in each lymph node. This reticular cell network distributes key molecules and provides a structure for immune cells to move around on. During infections, the network can suffer damage. A new study has now investigated the network’s structure in detail, using methods from graph theory. The study showed that the network is remarkably robust to damage: it can still support immune responses even when half of the reticular cells are destroyed. This is a further important example of how network connectivity achieves tolerance to failure, a property shared with other important biological and nonbiological networks.

Lymph nodes are critical sites for immune cells to connect, exchange information, and initiate responses to foreign invaders. More than 90% of the cells in each lymph node—the T and B lymphocytes of the adaptive immune system—only reside there temporarily and are constantly moving around as they search for foreign substances (antigen). When there is no infection, T and B cells migrate within distinct regions. But lymph node architecture changes dramatically when antigen is found, and an immune response is mounted. New blood vessels grow and recruit vast numbers of lymphocytes from the blood circulation. Antigen-specific cells divide and mature into “effector” immune cells. The combination of these two processes—increased influx of cells from outside and proliferation within—can make a lymph node grow 10-fold within only a few days [1]. Accordingly, the structural backbone supporting lymph node function cannot be too rigid; otherwise, it would impede this rapid organ expansion. This structural backbone is provided by a network of fibroblastic reticular cells (FRCs) [2], which secrete a form of collagen (type III alpha 1) that produces reticular fibers—thin, threadlike structures with a diameter of less than 1 μm. Reticular fibers cross-link and form a spider web–like structure. The FRCs surrounding this structure form the reticular cell network (Fig 1), which was first observed in the 1930s [3]. Interestingly, experiments in which the FRCs were destroyed showed that the collagen fiber network remained intact [4].

**Fig 1:**
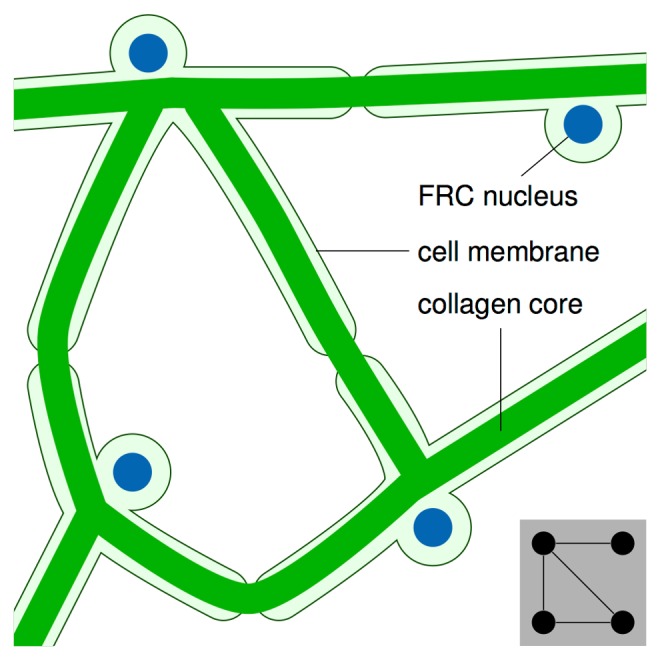
Structure of the reticular cell network. The reticular cell network is formed by fibroblastic reticular cells (FRCs) whose cell membranes ensheathe a core of collagen fibers that acts as a conduit system for the distribution of small molecules [5]. In most other tissues, collagen fibers instead reside outside cell membranes, where they form the extracellular matrix. Inset: graph structure representing the FRCs in the depicted network as “nodes” (circles) and the direct connections between them as “edges” (lines). Shape and length of the fibers are not represented in the graph.

Reticular cell networks do not only support lymph node structure; they are also important players in the immune response. Small molecules from the tissue environment or from pathogens, such as viral protein fragments, can be distributed within the lymph node through the conduit system formed by the reticular fibers [5]. Some cytokines and chemokines that are vital for effective T cell migration—and the nitric oxide that inhibits T cell proliferation [6]—are even produced by the FRCs themselves. Moreover, the network is thought of as a “road system” for lymphocyte migration [7]: in 2006, a seminal study found that lymphocytes roaming through lymph nodes were in contact with network fibers most of the time [8]. A few years before, it had become possible to observe lymphocyte migration in vivo by means of two-photon microscopy [9]. Movies from these experiments strikingly demonstrated that individual cells were taking very different paths, engaging in what appeared to be a “random walk.” But these movies did not show the structures surrounding the migrating cells, which created an impression of motion in empty space. Appreciating the role of the reticular cell network in this pattern of motion [8] suggested that the complex cell trajectories reflect the architecture of the network along which the cells walk.

Given its important functions, it is surprising how little we know about the structure of the reticular cell network—compared to, for instance, our wealth of knowledge on neuron connectivity in the brain. In part this is because the reticular cells are hard to visualize. In vivo techniques like two-photon imaging do not provide sufficient resolution to reliably capture the fine-threaded mesh. Instead, thin tissue sections are stained with fluorescent antibodies that bind to the reticular fibers and are imaged with high-resolution confocal microscopy to reveal the network structure. One study [10] applied this method to determine basic parameters such as branch length and the size of gaps between fibers. Here, we discuss a recent study by Novkovic et al. [11] that took a different approach to investigating properties of the reticular cell network structure: they applied methods from graph theory.

Graph theory is a classic subject in mathematics that is often traced back to Leonhard Euler’s stroll through 18th-century Königsberg, Prussia. Euler could not find a circular route that crossed each of the city’s seven bridges exactly once, and wondered how he could prove that such a route does not exist. He realized that this problem could be phrased in terms of a simple diagram containing points (parts of the city) and lines between them (bridges). Further detail, such as the layout of city’s streets, was irrelevant. This was the birth of graph theory—the study of objects consisting of points (nodes) connected by lines (edges). Graph theory has diverse applications ranging from logistics to molecular biology. Since the beginning of this century, there has been a strong interest in applying graph theory to understand the structure of networks that occur in nature—including biological networks, such as neurons in the brain, and more recently, social networks like friendships on Facebook. Various mathematical models of network structures have been developed in an attempt to understand network properties that are relevant in different contexts, such as the speed at which information spreads or the amount of damage that a network can tolerate before breaking into disconnected parts. Three well-known network topologies are random, small-world, and scale-free networks (Box 1). Novkovic et al. modeled reticular cell networks as graphs by considering each FRC to be a node and the fiber connections between FRCs to be edges (Fig 1).

**Box 1. Graph Theory and the Robustness of Real Networks**

After the publication of several landmark papers on network topology at the end of the previous century, the science of complex networks has grown explosively. One of these papers described “small-world” networks [16] and demonstrated that several natural networks have the amazing property that the average length of shortest paths between arbitrary nodes is unexpectedly small (making it a “small world”), even if most of the network nodes are clustered (that is, when neighbors of neighbors tend to be neighbors). The Barabasi group published a series of papers describing “scale-free” networks [17,18] and demonstrated that scale-free networks are extremely robust to random deletions of nodes—the vast majority of the nodes can be deleted before the network falls apart [15]. In scale-free networks, the number of edges per node is distributed according to a power law, implying that most nodes have very few connections, and a few nodes are hubs having very many connections. Thus, the topology of complex networks can be scale-free, small-world, or neither, such as with random networks [19]. Novkovic et al. [11] describe the clustering of the edges of neighbors and the average shortest path–length between arbitrary nodes, finding that reticular cell networks have small-world properties. Whether or not these networks have scale-free properties is not explicitly examined in the paper, but given that they are embedded in a three-dimensional space, that they “already” lose functionality when about 50% of the FRCs are ablated, and that the number of connected protrusions per FRC is not distributed according to a power law (see the data underlying their Figure 2g), reticular cell networks are not likely to be scale-free. Thus, the enhanced robustness of reticular cell networks is most likely due to their high local connectivity: Networks lose functionality when they fall apart in disconnected components, and high clustering means that the graph is unlikely to split apart when a single node is removed, because the neighbors of that node tend to stay connected [14]. Additionally, since the reticular cell network has a spatial structure (unlike the internet or the Facebook social network), its high degree of clustering is probably due to the preferential attachment to nearby FRCs when the network develops, which agrees well with Novkovic et al.’s recent classification as a small-world network with lattice-like properties [11].

Some virus infections are known to damage reticular cell networks [12], either through infection of the FRCs or as a bystander effect of inflammation. It is therefore important to understand to what extent the network structure is able to survive partial destruction. Novkovic et al. first approached this question by performing computer simulations, in which they randomly removed FRC nodes from the networks they had reconstructed from microscopy images. They found that they had to remove at least half of the nodes to break the network apart into disconnected parts. To study the effect of damage on the reticular cell network in vivo rather than in silico, Novkovic et al. used an experimental technique called conditional cell ablation. In this technique, a gene encoding the diphtheria toxin receptor (DTR) is inserted after a specific promoter that leads it to be expressed in a particular cell type of interest. Administration of diphtheria toxin kills DTR-expressing cells, leaving other cells unaffected. By expressing DTR under the control of the FRC-specific *Ccl19* promoter, Novkovic et al. were able to selectively destroy the reticular cell network and then watch it grow back over time. Regrowth took about four weeks, and the resulting network properties were no different from a network formed naturally during development. Thus, it seems that the reticular cell network structure is imprinted and reemerges even after severe damage. This finding ties in nicely with previous data from the same group [13], showing that reticular cell networks form even in the absence of lymphotoxin-beta receptor, an otherwise key player in many aspects of lymphoid tissue development. Together, these data make a compelling case that network formation is a robust fundamental trait of FRCs.

Next, Novkovic et al. varied the dose of diphtheria toxin such that only a fraction of FRCs were destroyed, effectively removing a random subset of the network nodes. They measured in two ways how FRC loss affects the immune system: they tracked T cell migration using two-photon microscopy and they determined the amount of antiviral T cells produced by the mice after an infection. Remarkably, as predicted by their computer simulations, lymph nodes appeared capable of tolerating the loss of up to half of FRCs with little effect on either T cell migration or the numbers of activated antiviral T cells. Only when more than half of the FRCs were destroyed did T cell motion slow down significantly and the mice were no longer able to mount effective antiviral immune responses. Such a tolerance of damage is impressive—for comparison, consider what would happen if one were to close half of London’s subway stations!

Robustness to damage is of interest for many different networks, from power grids to the internet [14]. In particular, the “scale-free” architecture that features rare, strongly connected “hub” nodes is highly robust to random damage [15]. Novkovic et al. did not address whether the reticular cell network is scale-free, but it is likely that it isn’t (Box 1). Instead, the network’s robustness probably arises from its high degree of clustering, which means that the neighbors of each node are likely to be themselves also neighbors. If a node is removed from a clustered network, then there is still likely a short detour available by going through two of the neighbors. Therefore, one would have to randomly remove a large fraction of the nodes before the network structure breaks down. High clustering in the network could be a consequence of the fact that multiple fibers extend from each FRC and establish connections to many FRCs in its vicinity. A question not yet addressed by Novkovic et al. is how robust reticular cell networks would be to nonrandom damage, such as a locally spreading viral infection. In fact, scale-free networks are drastically more vulnerable to targeted rather than random damage: the United States flight network can come to a grinding halt by closing a few hub airports [15]. Less is known about the robustness to nonrandom damage for other network architectures, and the findings by Novkovic et al. motivate future research in this direction.

Novkovic et al. did not yet explicitly identify all mechanisms that hamper T cell responses when more than half of the FRCs are depleted. But given the reticular cell network’s many different functions, this could occur in several ways. For instance, severe depletion might prevent the secretion of important molecules, halt the migration of T cells, prevent the anchoring of antigen-presenting dendritic cells (DCs) on the network, or cause structural disarray in the tissue. In addition to the effects on T cell migration, Novkovic et al. also showed that the amount of DCs in fact decreased when FRCs were depleted, emphasizing that several mechanisms are likely at play. Disentangling these mechanisms will require substantial additional research efforts.

The current reticular cell network reconstruction by Novkovic et al. is based on thin tissue slices. It will be exciting to study the network architecture when it can be visualized in the whole organ. Some aspects of the network may then look different. For instance, those FRCs that are near the border of a slice will have their degree of connectivity underestimated, as not all of their neighbors in the network can be seen. Further refinements of the network analysis may also consider that reticular fibers are real physical objects situated in a three-dimensional space (unlike abstract connections such as friendships). Migrating T cells may travel quicker via a short, straight fiber than on a long, curved one, but the network graph does not make this distinction. More generally, it would be interesting to understand conceptually how reticular cell networks help foster immune cell migration. While at first it appears obvious that having a “road system” should make it easier for cells to roam lymph node tissue, three different theoretical studies have in fact all concluded that effective T cell migration should also be possible in the absence of a network [20–22]. A related question is whether T cells are constrained to move only on the network or are merely using it for loose guidance. For instance, could migrating T cells be in contact with two or more network fibers at once or with none at all? This would make the relationship between cell migration and network structure more complex than the graph structure alone suggests.

There is also some evidence that T cells can migrate according to what is called a Lévy walk [23]—a kind of random walk where frequent short steps are interspersed with few very long steps, a search strategy that appears to occur frequently in nature (though this is debated [24]). While there is so far no evidence that T cells perform a Lévy walk when roaming the lymph node [25], this may be in part due to limitations of two-photon imaging, and one could speculate that reticular cell networks might in fact be constructed in a way that facilitates this or another efficient kind of “search strategy.” Resolving this question will require substantial improvements in imaging technology, allowing individual T cells to be tracked across an entire lymph node.

No doubt further studies will address these and other questions, and provide further insights on how reticular cell networks benefit immune responses. Such advances may help us design better treatments against infections that damage the network. It may also help us understand how we can best administer vaccines or tumor immune therapy treatments in a way that ensures optimal delivery to immune cells in the lymph node. As is nicely illustrated by the study of Novkovic et al., mathematical methods may well play key roles in this quest.

## Abbreviations

DC: dendritic cell
DTR: diphtheria toxin receptor
FRC: fibroblastic reticular cell
